# Sleep Quality, Relationship Functioning, and Health-Related Quality of Life in Partners of CPAP-Treated OSA Patients

**DOI:** 10.32872/cpe.20295

**Published:** 2026-08-31

**Authors:** Giulia Marafioti, Enrica S. Vinci, Laura Culicetto, Viviana Lo Buono, Giovanni L. Cipriano, Rosalia Silvestri, Amelia Rizzo

**Affiliations:** 1 IRCCS Bonino-Pulejo Neurolesi Center, Messina, Italy; 2 National Institute of Social Welfare, Medical Legal Center of Messina, Messina, Italy; 3 Sleep Medicine Center, UOSD of Neurophysiopathology and Movement Disorders, Department of Clinical and Experimental Medicine, University of Messina, Messina, Italy; Philipps-University of Marburg, Marburg, Germany

**Keywords:** obstructive sleep apnea, CPAP, sleep quality, couple relationship, quality of life, partner

## Abstract

**Background:**

Obstructive Sleep Apnea Syndrome (OSAS) is a chronic condition that negatively affects not only the patient but also the partner, influencing their sleep quality, psychological well-being, and couple relationship. However, scientific literature has given little attention to the partner's experience, especially regarding the impact of Continuous Positive Airway Pressure (CPAP) treatment on their partners’ health. The aim of the present study is to investigate sleep quality, couple relationship quality, and quality of life (QoL) in Italian-speaking partners of patients with OSAS treated with CPAP, compared to partners of healthy individuals.

**Method:**

The study involved 100 participants, with a final sample of 48 partners of OSA patients and 49 partners of healthy subjects as a control group, assessed using the Pittsburgh Sleep Quality Index (PSQI), the Dyadic Adjustment Scale (DAS), and the SF-36 Health Survey. Statistical analysis included independent samples *t*-tests and Pearson’s correlations.

**Results:**

Partners of patients with OSAS reported significantly poorer sleep quality (PSQI), lower levels of dyadic consensus and cohesion (DAS), and reduced perception of physical and mental health (SF-36) compared to the control group. Correlations revealed that sleep quality was negatively associated with relationship quality and perceived health, while years of relationship were positively associated with affection and cohesion, but negatively with sleep quality.

**Conclusions:**

The findings confirm the interconnection between sleep quality and relationship functioning. A clinical approach that includes the partner in OSA treatment pathways may improve the overall well-being of the dyad.

## Highlights

- Partners of patients with Obstructive Sleep Apnea (OSA) report poorer sleep, health, and relationship quality.
- OSA is a "shared" disorder that negatively impacts couple cohesion and consensus.
- Poor sleep quality in partners is linked to worse relationship functioning and health.
- A dyadic approach to OSA treatment may improve overall well-being for the couple.

Obstructive Sleep Apnea Syndrome (OSAS) falls within the category of Sleep-Disordered Breathing (SDB). SDBs are frequently comorbid with various pathologies; for example, they are common in patients with heart failure (Khokhrina et al., 2022). Moreover, they have been epidemiologically and pathophysiologically linked to ischemic heart disease, atrial fibrillation, arterial hypertension (Fava et al., 2011; Gonzaga et al., 2015; Gopalakrishnan & Tak, 2011), diabetes mellitus, metabolic syndrome (Drager et al., 2013), sudden death (Gami et al., 2005), and stroke (Yaggi et al., 2005). The prevalence of SDB is progressively increasing due to the rising incidence of obesity (Peppard et al., 2013). In this context, it is essential to distinguish between two primary syndromes: Obstructive Sleep Apnea (OSA) and Central Sleep Apnea (CSA). OSAS is a widespread but often unrecognized respiratory disorder characterized by episodes of partial or complete upper airway closure during sleep (Locke et al., 2022).

The complications of this syndrome frequently affect the cardiovascular system (hypertension initially appearing at night and later during the day) or the nervous system, leading to cognitive and mood disorders (depression, anxiety, emotional instability; Harding, 2000). These complications strongly impact not only the life of the affected individual but also that of their partner, often leading to imbalances in the relationship. Several studies in the literature have highlighted how sleep disorders or sleep deprivation can negatively affect cognitive, relational, and emotional processes, consequently influencing interpersonal interpretation abilities (Durmer & Dinges, 2005; Hasler & Troxel, 2010; Troxel et al., 2007). Individuals affected by OSAS are often considered ‘heavy snorers’. Various studies emphasize the great difficulty that partners experience in managing this condition (Cartwright, 2008; Luyster et al., 2016). Many partners report feeling deeply distressed while witnessing these nocturnal apneas (Troxel et al., 2009). On one hand, continuous positive airway pressure treatment often reduces snoring and apneic events, which are among the main causes of sleep disruption for partners of individuals with OSAS. In this sense, CPAP can significantly improve the partner’s sleep continuity and perceived restfulness. On the other hand, some partners may initially experience sleep disturbance due to device-related factors such as machine noise, airflow sounds, mask leaks, or the physical presence of the equipment in bed. For certain couples, especially in the early adaptation phase, these elements may temporarily disrupt sleep, with possible repercussions on the couple’s relationship quality.

However, in the literature, few and relatively outdated studies have explored the association between couple quality and sleep quality. Additionally, the results are conflicting: some studies reveal a clear deterioration in relationship quality when a sleep disorder is present (McArdle et al., 2001; Virkkula et al., 2005), whereas others do not confirm such an association (Broström et al., 2010; Parish & Lyng, 2003). Further research has investigated how OSAS is a shared problem affecting both spouses, leading to fragmented sleep, daytime sleepiness, and reduced quality of life (QoL), often resulting in tense marital relationships (Doherty et al., 2003; Henry, 2016) found a significant association between chronic insomnia and the deterioration of romantic relationships. Pankhurst and Home (1994) were the first to analyze the effects of nocturnal movements of OSAS patients on their partners' sleep in couples without other sleep disorders. A gender-based study showed that men report more nighttime movements than women, and women are more likely to experience sleep disturbances caused by their partner compared to men (Baron et al., 2009). Some qualitative studies have highlighted the importance of partners' perspectives in understanding the experience of OSAS. Improvements in sleep and increased alertness in both individuals involved contribute to a better understanding of the health consequences of OSAS. The nighttime and daytime consequences of OSAS often lead individuals to sleep in separate beds, reducing intimacy and contributing to relationship tensions. This is supported by various qualitative and quantitative studies that report poor sleep quality and impaired QoL, affecting both OSA patients and their partners (Baron et al., 2011; Hoy et al., 1999). Conversely, some cross-sectional studies have demonstrated adverse associations between OSAS and relationship quality as reported by the patient or partner (McArdle et al., 2001; Virkkula et al., 2005). It is well known that QoL improves after CPAP treatment; however, the effects on the patient's bed partner have received little attention. The role of relationship factors in CPAP adherence remains poorly understood, but some studies suggest that partners may have both positive and negative influences on CPAP adoption and use. In this regard, some studies have identified that partners of OSAS patients also benefit from treatment, showing improved perception levels, QoL (Billmann & Ware, 2002), sleep quality (Luyster et al., 2016), and couple relationship quality (Beninati et al., 1999). Specifically, a study conducted at Rush University Medical Center in Chicago examined the sleep of married couples, recording it in a laboratory before and after the husband's CPAP treatment to assess adherence levels. The study found that adherence was strongly correlated with the wives' involvement, emphasizing that bed-sharing is fundamental for improving treatment adherence (Cartwright, 2008). Based on the literature and previous research, this study aims to address the Research Objectives (RO):

- **RO 1** – Compare Sleep Quality, Relationship Functioning, and Health-Related Quality of Life in Partners of Patients with and without CPAP-Treated OSAS;
- **RO 2** – Compare differences in correlational patterns of Sleep Quality, Relationship Functioning, and Health-Related Quality of Life in Partners of patients with and without CPAP-Treated OSAS.

The impact of OSAS extends beyond the individual patient, significantly affecting the partner's sleep quality, psychological well-being, and relationship dynamics. The literature highlights the distress experienced by partners due to sleep disruptions, emotional strain, and reduced intimacy, often leading to tensions within the couple. However, findings remain inconsistent, with some studies confirming a decline in relationship satisfaction, while others do not observe such an association. CPAP therapy has been shown to improve sleep quality and overall well-being for both patients and their partners, yet its effects on relationship satisfaction and psychological health are still underexplored. The present study aims to bridge this gap by comparing sleep quality, couple satisfaction, and QoL in partners of people with and without CPAP treatment (RO1) and investigating differences in correlational patterns when compared to a healthy control group (RO2). By addressing these aspects, this research seeks to provide deeper insights into the broader implications of OSAS and highlight the importance of partner involvement in treatment adherence and overall well-being.

## Materials and Method

The study protocol initially included the administration of an informed consent form, a document that thoroughly informed participants about the study and collected their authorization to participate. Subsequently, each participant completed a demographic form to collect information on demographics, education level, any pathologies, and comorbidities. The questionnaires were administered individually once a week, under the supervision of a psychologist who provided clarifications and support to the participants, if needed. This protocol ensured the accurate collection of data and ensured that participants had a clear understanding of the questions and the process of participating in the study.

### Participants

A total of 100 subjects were recruited in the research, divided into two groups: 50 partners of OSA patients (see Table 1 for the sample characteristics), attending the Sleep Medicine Center of the University Hospital of Messina "G. Martino," between October 2019 and March 2020, and 50 partners of healthy individuals as a control group.

Participants were classified into two groups based on clinical status: individuals diagnosed with OSA and healthy controls (HC). OSA patients were routinely monitored for CPAP treatment adherence through regular clinical follow-up and device-recorded data.

Inclusion in the OSA group required a confirmed clinical diagnosis established through standard polysomnographic assessment and medical evaluation conducted by a sleep specialist. Only adults were included, and participants had to be in a stable romantic relationship, given the relational variables investigated in the study. Inclusion in the HC group required the absence of a diagnosis of sleep disorders and the absence of self-reported symptoms suggestive of sleep apnea, as well as the same relational status criteria applied to the OSA group.

Exclusion criteria for both groups included the presence of severe psychiatric disorders, neurodegenerative conditions, major uncontrolled medical illnesses, or cognitive impairments that could interfere with comprehension of the questionnaires. Individuals with current substance abuse or other diagnosed sleep disorders were also excluded. For the HC group specifically, any previous diagnosis of OSA or other clinically relevant sleep disturbances represented an exclusion criterion.

Two participants from the OSA partners group were excluded due to dropout prior to protocol completion. One participant from the HC group was excluded because they were not co-sleeping with their partner. In accordance with the predefined inclusion and exclusion criteria, the final sample consisted of 48 partners of individuals diagnosed with OSA and 49 HC group.

Participants were recruited using a non-probability sampling method. The OSA group was likely selected through consecutive sampling from patients attending a sleep clinic or undergoing diagnostic evaluation, while the HC group was recruited through convenience sampling from the community, ensuring comparability in basic sociodemographic characteristics. This sampling strategy aimed at obtaining two clinically distinct but demographically comparable groups rather than a randomly selected population sample.

### Instruments

#### Pittsburgh Sleep Quality Index (PSQI)

The PSQI is a self-report questionnaire developed by researchers at the University of Pittsburgh (Buysse et al., 1989). It is intended to be a standardized sleep questionnaire that researchers and doctors can easily use, and it is utilized across various populations and in different contexts, including research activities and clinical settings. It consists of 19 items that assess the subjects’ perceived sleep quality and disturbances over a 1-month period, and completion takes between 5 and 10 minutes (Buysse et al., 1989). The 19 items are grouped into 7 composite items, rated on a scale from 0 to 3, which, when summed, give the overall PSQI score. The overall score ranges from 0 to 21, where "≤ 5" corresponds to good sleep quality while "> 5" corresponds to poor sleep quality. These 7 composite items explore sleep quality through a wide range of domains that include: The usual methods of awakening, the duration of sleep, sleep latency, the frequency and severity of specific problems that arise during sleep (such as the presence of pain, frequent urination, breathing difficulties, the presence of snoring, dream activity, temperature), the use of hypnotic medications, daytime disturbances, and usual effectiveness. All of this is measured retrospectively.

The PQSI exhibits good psychometric characteristics to the extent that it can be said that the authors have effectively developed a reliable, valid, and standardized measure of sleep quality. In addition to the promising reliability and validity of the measure, its brevity and accessibility as a free measure give it great potential for clinical practice (Buysse et al., 1989). In the present study, the reliability of the Italian version of the PSQI was assessed using Cronbach’s alpha, which yielded values between .80 and .90, indicating good internal consistency of the scale.

#### Dyadic Adjustment Scale (DAS)

The DAS is a multidimensional tool designed by Spanier (1976). The DAS consists of 32 items divided into four subscales: Dyadic Consensus (DC); Dyadic Satisfaction (DS); Dyadic Cohesion (DH); Affective Expression (AE). The sum of the four scales provides a total score that expresses the overall level of agreement within the couple. The dyadic consensus scale (DC) consists of 13 items and assesses the degree of agreement and disagreement between partners on topics such as leisure time management and finances, or on religion, friendships, and household organization. The dyadic satisfaction scale (DS) consists of 10 items and evaluates the happiness or unhappiness that couples perceive regarding their relationship; this scale observes the frequency of arguments, the pleasure or lack thereof in being together, and the consideration of separation or divorce. The diadic cohesion scale (DH) consists of 5 items and evaluates the amount of time partners share pleasant activities such as social interests, dialogue, or having common goals. Finally, the affective expression scale (AE) consists of 4 items and evaluates how the couple expresses their feelings, love, and sexuality. It is a simple tool that takes about 6-7 minutes to complete; a quiet place, a pen, and the questionnaire are required. The scale is aimed at both married and unmarried couples, and it is filled out independently. The instruction given to the subjects is to carefully read the instructions and understand their meaning. It is considered the most widely used measurement tool for evaluating the "quality" of the relationship (Spanier, 1976). In the present study, the reliability of the Italian versions of the DAS was assessed using Cronbach’s alpha, yielding values between .80 and .90 for both scales, indicating good internal consistency.

Total scores on the scale range from 0 to 150. Higher scores reflect greater relationship quality, satisfaction, and adjustment, whereas lower scores indicate relational dissatisfaction or distress. In his original validation study, Spanier (1976) proposed a clinical cutoff whereby scores below 101 indicate *relational distress*, and scores of 102 or higher indicate a *non-distressed* relationship. For reference, Spanier reported mean total scores of 70.7 in a divorced sample and 114.8 in a married sample, supporting the discriminative measures’ validity (Spanier, 1976).

#### Health Survey (SF-36)

The Short Form-36 Health Survey (SF-36; Ware & Sherbourne, 1992) was used to assess participants' perceived health status. The instrument evaluates eight domains: Physical Functioning, Role Physical, Bodily Pain, General Health, Vitality, Social Functioning, Role Emotional, and Mental Health. Two summary measures can also be derived: the Physical Component Summary (PCS) and the Mental Component Summary (MCS). The Italian validation by Apolone and Mosconi (1998) demonstrated good psychometric properties, with Cronbach’s alpha coefficients for the subscales ranging from 0.73 to 0.93, indicating satisfactory to excellent internal consistency.

#### Statistical Analysis

All statistical analyses were conducted with SPSS (Version 27.0). Independent-samples *t* tests were used to compare Sleep Quality, Relationship Functioning, and Health-Related QoL between partners of patients with CPAP-treated OSA and partners of individuals without OSA. Pearson’s correlation coefficients (*r*) were computed separately within each group to examine associations among the three constructs. Differences in correlational patterns between the two groups were evaluated using Fisher’s *r* to *z* transformation. For each analysis, unadjusted p-values were reported, and results were interpreted according to the conventional criterion of *p* < .05, without applying additional corrections for multiple comparisons. Effect sizes (Cohen’s *d* for group differences and *r* for correlations) and 95% confidence intervals were calculated to aid interpretation of the findings.

## Results

### Sample Characteristics

The data analysis began with the qualitative analysis of various constructs. Table 1 presents the sociodemographic and relationship characteristics of the OSA (*n* = 48) and HC (*n* = 49) partner groups. All participants included in the dyadic analyses reported being currently involved in a stable romantic relationship. The category “in a stable relationship / partnered” includes not only individuals who are unmarried or cohabiting, but also those with other civil statuses (e.g., separated or widowed) who currently have a partner.

**Table 1:** Sociodemographic and Relationship Characteristics of the HC and OSA Groups

Variable / Category	HC *n* (%)	OSA *n* (%)
Number of valid protocols		
Valid	48 (96.0%)	50 (100.0%)
Invalid	2 (4.0%)	0 (0.0%)
Civil/marital status		
In a stable relationship / partnered	47 (97.9%)	49 (98.0%)
Other civil status (e.g., separated/widowed), with current partner	1 (2.1%)	1 (2.0%)
Relationship duration (years)		
< 1 year	0 (0.0%)	1 (2.0%)
1–3 years	3 (6.3%)	1 (2.0%)
4–6 years	2 (4.2%)	3 (6.0%)
7–10 years	2 (4.2%)	2 (4.0%)
11–15 years	9 (18.8%)	5 (10.0%)
16–20 years	15 (31.3%)	4 (8.0%)
21–25 years	7 (14.6%)	16 (32.0%)
> 25 years	10 (20.8%)	18 (36.0%)
Educational level		
Primary school	2 (4.2%)	7 (14.0%)
Secondary school	11 (22.9%)	21 (42.0%)
High school diploma	26 (54.2%)	12 (24.0%)
University degree	9 (18.8%)	10 (20.0%)
Sleeping with Partner		
Yes	48 (100.0%)	49 (98.0%)
No	0 (0.0%)	1 (2.0%)

*Note.* Statistics on the whole sample (*N* = 100), valid cases for OSA = Obstructive Sleep Apnea partners (*n* = 49), and for Healthy Controls (*n* = 48).

Given that the DAS assesses current couple functioning, DAS scores, DAS-based classifications, and correlations involving DAS variables were computed only for participants who reported having a current romantic partner. Participants not sharing the bed with their partner were excluded from dyadic analyses, despite reporting an ongoing romantic relationship; to this extent, co-sleeping status was described separately and was not used as a proxy for relationship status.

The OSA group shows a higher concentration in the longest relationship categories (> 20 years), while the HC group appears more in the mid-range durations (11 to 20 years). Educationally, the HC group is more represented in the high school category, whereas the OSAS group shows a greater proportion with lower secondary education. All participants in the HC group reported sleeping with their partner, whereas 49 out of 50 participants in the OSA group reported co-sleeping. Therefore, the participant who did not meet the inclusion criteria was excluded from the analysis.

### RO 1 – Sleep Quality, Relationship Functioning, and Health-Related Quality of Life in Partners of Patients With and Without CPAP-Treated OSA

To address Research Objective 1 (RO1), Student’s *t*-test for independent samples was performed (see Table 2) to explore whether the presence of OSA, even when treated with continuous positive airway pressure (CPAP), is associated with differences in partners’ subjective sleep experience, relational dynamics, and perceived well-being.

**Table 2:** Mean Scores and Standard Deviations Obtained by the Participants

Measure / Group	*M*	*SD*
SF-36 PCS		
OSA Partners	63.23*	19.73
HC Partners	75.57*	10.85
SF-36 MCS		
OSA Partners	65.37*	16.34
HC Partners	74.77*	10.44
PSQI		
OSA Partners	11.06*	3.69
HC Partners	6.00*	3.37
Dyadic Consensus (DAS)		
OSA Partners	3.06*	0.74
HC Partners	4.07*	0.59
Dyadic Satisfaction (DAS)		
OSA Partners	3.67	0.62
HC Partners	3.64	0.68
Affective Expression (DAS)		
OSA Partners	2.27	0.53
HC Partners	2.15	0.46
Dyadic Cohesion (DAS)		
OSA Partners	1.04*	1.03
HC Partners	2.97*	0.98

*Note*. DAS = Dyadic Adjustment Scale; PCS = Physical Component Score; MCS ***=*** Mental Component Score; PSQI = Pittsburgh Sleep Quality Index; HC = Healthy Controls; OSA Partners = Obstructive Sleep Apnea partners.

*p < .05.

The data analysis conducted highlighted some statistically significant differences. Specifically, in the control group, there is a greater perception of physical health status compared to the group of partners with OSA patients, *t*(95) = -3.80, *p <* .001, and this result is also confirmed regarding the perception of mental health status, *t*(95) = -3.80, *p =* .001. Again, regarding sleep quality, the group of partners of OSA patients reports poorer sleep quality compared to the control group, *t*(95) = 0.85, *p =* .032. Subsequently, we considered the construct of couple functioning. Specifically, in the dimension of dyadic consensus, the control group records higher levels of agreement within the couple, *t*(95) = -0.48, *p =* .042, compared to the group of partners of patients with OSA. No significant differences were found in the dimensions of dyadic satisfaction, emotional expression, and cohesion.

In other words, partners of patients with OSA seem to experience a tangible impact on their personal well-being, both physically and psychologically, and they also report poorer sleep. However, when the focus shifts to the quality of the relationship itself, the differences are more nuanced.

Specifically, the lower dyadic consensus suggests that these couples may experience more difficulties in agreement or coordination in everyday matters. Yet, the absence of differences in satisfaction, emotional expression, and cohesion indicates that the emotional bond and overall relationship satisfaction are not necessarily compromised. In essence, OSA appears to affect well-being and certain functional aspects of the relationship more than its emotional core.

We first examined the overall level of couple functioning in the total sample. Results showed that 37% of couples fell within the functional range (DAS total score range 99-113), indicating high harmony and good relational adjustment. The majority, 57%, were classified in the functional distress range (71–98), suggesting the presence of relational strain but with the capacity to manage conflicts constructively. The remaining 6% (0–70) were characterized by marked relational dysfunction, reflecting persistent disagreement and poor conflict resolution.

**Figure 1:**
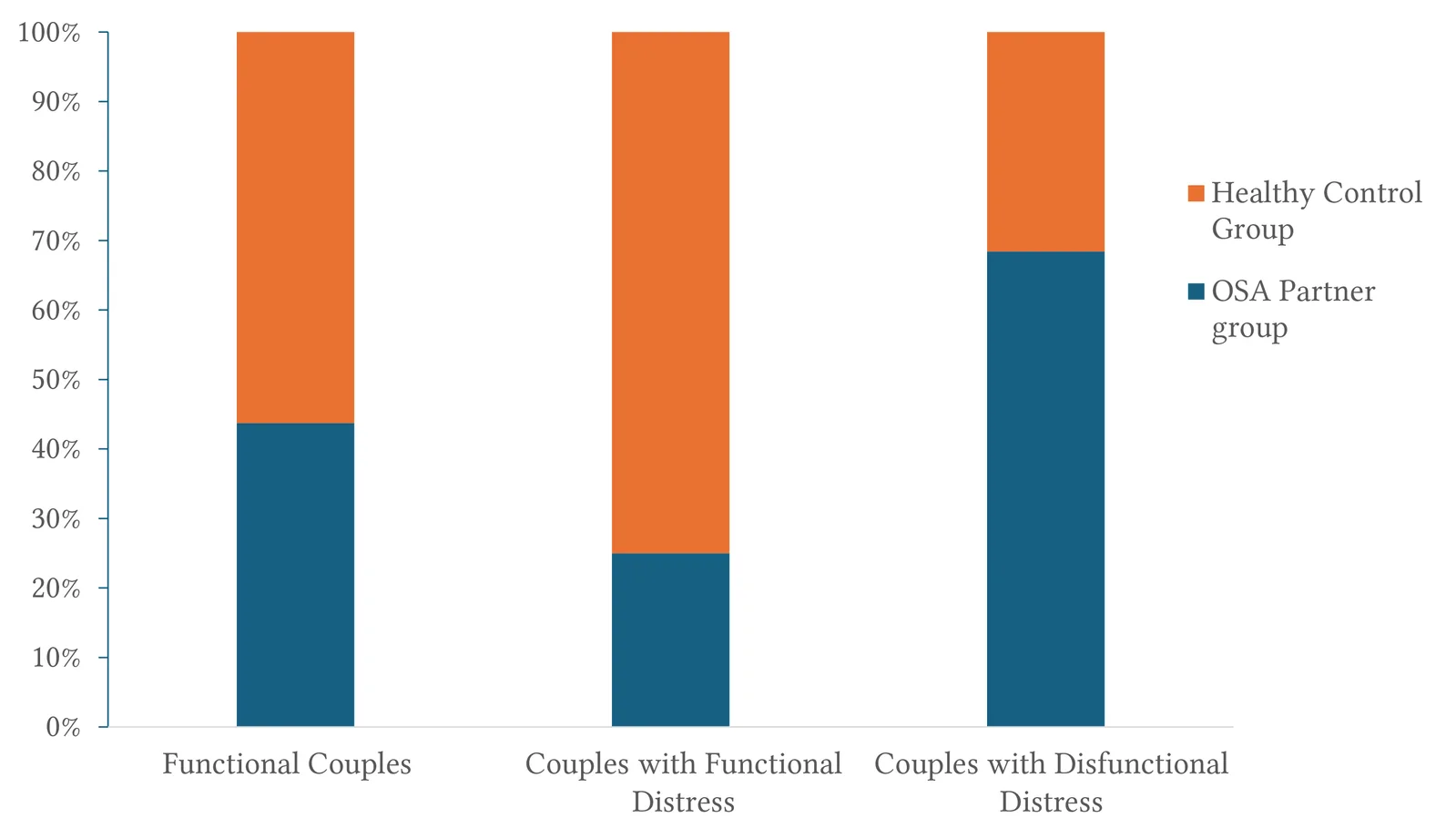
Comparison of Quality of Couple Relationship *Note.* OSA Partners = Obstructive Sleep Apnea partners.

We then compared the two groups. Among partners of patients with OSA, 59.2% of couples were classified as functional, 38.8% as experiencing dysfunctional distress, and 2% as functional distress. In contrast, the control group showed a higher proportion of functional relationships (76.3%), a lower percentage of dysfunctional distress (17.4%), and 6.3% in the functional distress range. These differences are illustrated in Figure 1.

Sleep quality was categorized according to the standard PSQI global cut-off (> 5) commonly used to distinguish poor from good sleepers (Buysse et al., 1989). Importantly, good sleep quality was not defined as an a priori inclusion criterion for the control group; rather, controls were recruited based on the general eligibility criteria described in the Method section. The fact that the control group consisted exclusively of “good sleepers” therefore reflects the observed distribution in this sample, and not a selection rule. Accordingly, 0% of controls scored > 5 (poor sleepers), whereas 55% of partners of patients with OSA exceeded the cut-off. Given this unexpectedly homogeneous profile in controls, group differences in sleep quality should be interpreted cautiously, as the absence of poor sleepers may contribute to an overestimation of between-group differences. To contextualize these findings, we also compared the observed sleep quality to population-based normative evidence. In community samples, PSQI mean values are typically around ~ 4–5, and the proportion of individuals above the > 5 cut-off is commonly non-negligible (e.g., around one quarter in large population-based surveys; Hinz et al., 2017). In this respect, the control group in the present study appears to exhibit unusually good sleep quality relative to general population benchmarks, which further supports a cautious interpretation of group comparisons involving PSQI.

### RO 2 – Differences in Correlational Patterns of Sleep Quality, Relationship Functioning, and Health-Related Quality of Life in Partners of Patients With and Without CPAP-Treated OSA

In the data analysis, the next examination proceeded with the differences in correlational patterns. The table below reports the Pearson correlations with the *p*-value obtained by the participants (Table 3 and 4).

**Table 3:** Correlations Among Partners of Patients With CPAP-Treated OSA (n = 48)

Variable	1	2	3	4	5	6	7
1. DAS DC	–	.37**	.41**	.41**	.04	.04	.12
		*.009*	*.004*	*.004*	*.805*	*.765*	*.410*
2. DAS DS		–	.21	.32*	–.02	.09	.23
			*.144*	*.027*	*.892*	*.525*	*.112*
3. DAS AE			–	.18	.16	–.14	–.12
				*.217*	*.271*	*.322*	*.413*
4. DAS DH				–	–.01	.15	.09
					*.043*	*.309*	*.551*
5. PSQI					–	–.51**	–.49**
						*.000*	*.000*
6. SF-36 PCS						–	.81**
							*.000*
7. SF-36 MCS							–

*Note.* Shows Pearson Correlations and *p*-values. DAS DC = Dyadic Adjustment Scale Dyadic Consensus; DAS DS = Dyadic Adjustment Scale Dyadic Satisfaction; DAS AE = Dyadic Adjustment Scale Affective Expression; DAS DH = Dyadic Adjustment Scale Dyadic Cohesion; PSQI = Pittsburgh Sleep Quality Index; SF-36 PCS = Physical Component Summary; SF-36 MCS = Mental Component Summary.

Italics indicate *p*-values. *p < .05. **p < .01.

**Table 4:** Correlations Among Partners of Healthy Controls (n = 49)

Neuropsychological Assessment	1	2	3	4	5	6	7
1. DAS DC	–	.30*	.24	.31*	–.26	.15	.22
		*.036*	*.096*	*.031*	*.076*	*.303*	*.131*
2. DAS DS		–	.36*	.44**	–.39**	.33*	–.01
			*.013*	*.002*	*.006*	*.023*	*.964*
3. DAS AE			–	.52**	–.24	.31*	.25
				*.000*	*.104*	*.030*	*.085*
4. DAS DH				–	–.34*	.33*	.18
					*.017*	*.022*	*.217*
5. PSQI					–	–.31*	–.09
						*.035*	*.526*
6. SF-36 PCS						–	.42**
							*.003*
7. SF-36 MCS							–

*Note*. Shows Pearson Correlations and *p*-values. DAS DC = Dyadic Adjustment Scale Dyadic Consensus; DAS DS = Dyadic Adjustment Scale Dyadic Satisfaction; DAS AE = Dyadic Adjustment Scale Affective Expression; DAS DH = Dyadic Adjustment Scale Dyadic Cohesion; PSQI = Pittsburgh Sleep Quality Index; SF-36 PCS = Physical Component Summary; SF-36 MCS = Mental Component Summary.

Italics indicate *p*-values. *p < .05. **p < .01.

The correlation analysis revealed several meaningful relationships in partners of patients undergoing OSA treatment. Dyadic consensus was positively associated with satisfaction, cohesion, affective expression, and the length of the relationship, indicating that longer-lasting couples tend to show greater harmony, emotional expression, and overall relationship satisfaction. Dyadic satisfaction also increased with cohesion and relationship duration, while affective expression grew with the years together. Cohesion was positively related to relationship length but negatively correlated with sleep quality (PSQI), suggesting that poorer sleep reduces couple cohesion. Sleep quality itself was negatively associated with physical and mental health and, unexpectedly, with relationship duration, indicating that longer relationships may experience poorer sleep. Physical health (PCS) declined with longer relationship length, while mental health (MCS) decreased as sleep quality worsened, highlighting the interconnection between sleep, health perception, and relationship functioning.

In conclusion, in the OSA partner group, sleep quality was significantly associated with both physical and mental health but showed no correlation with relationship quality, suggesting that poor sleep primarily affects individual well-being rather than relational functioning. Conversely, in the healthy partner group, relationship quality was positively correlated with sleep quality and physical health, but not with mental health, indicating that for these partners, better relational functioning is linked to more restorative sleep and better perceived physical well-being, while mental health appears independent of relationship quality.

## Discussion

The findings of the present study suggest that partners of patients diagnosed with OSA face multiple challenges, including poorer sleep quality, lower perceived physical and mental health, and reduced levels of couple cohesion and dyadic consensus. While not all subscales of the DAS revealed statistically significant differences, the overall pattern aligns with previous literature highlighting that OSA is not solely a patient-centered condition but a “shared” disorder with significant repercussions for partners and the couple as a relational unit (Bercea et al., 2013; Schröder & O’Hara, 2005).

To minimize the possibility that these differences reflect preexisting characteristics rather than the effects of OSA and CPAP use, both groups were selected using similar inclusion criteria (adulthood, stable relationships, absence of severe psychiatric or neurological conditions), and statistical analyses controlled for potential confounders such as age, relationship duration, and educational level. Although this is an observational study, these precautions support the interpretation that differences in sleep quality, health, and couple functioning are consistent with the impact of OSA and CPAP adherence rather than innate couple characteristics.

Sleep quality emerged as a critical factor. Partners of OSA patients reported disrupted sleep, which was significantly associated with poorer physical and mental health but not directly with relationship quality. This is consistent with evidence that even mild or intermittent nocturnal disturbances, such as snoring or apneic events, can impair partner sleep and increase stress and interpersonal conflict (Luyster et al., 2016). In contrast, in the control group, relationship quality correlated with sleep quality and physical health, but not mental health, suggesting that for healthy partners, relational functioning is closely tied to restorative sleep and physical well-being, while mental health remains relatively independent.

Despite CPAP therapy being well established for improving outcomes in patients with OSA (McArdle et al., 1999), its effects on partners’ well-being and couple functioning are less understood. The current findings tentatively support the idea that partner involvement in CPAP adherence may have broader benefits beyond patient health, potentially enhancing relational functioning, although longitudinal or intervention studies are needed to confirm this (Luyster et al., 2016).

Using preliminary classifications, couples were categorized as functional, experiencing functional distress, or dysfunctional. The majority (57%) fell into functional distress, characterized by conflict alongside the ability to resolve disagreements. A smaller proportion demonstrated persistent conflict (dysfunctional distress), while 37% were fully functional. Partners of OSA patients were more likely to experience dysfunctional distress (38%) than controls (17%), while functional relationships were more common in the control group (76%), suggesting greater cohesion and relational stability in healthy couples. These findings echo prior research linking chronic illness to increased relational strain (Luyster et al., 2016), though sampling characteristics may partially influence these differences. It is important to consider that the patient couples (PC) included in this study may represent a subset of partnerships that have endured despite the challenges posed by sleep apnea. Prior to treatment, these couples could have experienced more severe disruptions in relational functioning, including increased conflict, emotional distancing, or diminished cohesion.

Correlational analyses provided further insights. In partners of OSA patients, dyadic consensus correlated positively with satisfaction, cohesion, affective expression, and relationship duration, suggesting that longer relationships may sustain higher relational quality and emotional expression. However, cohesion was negatively correlated with sleep quality (PSQI), and sleep quality itself correlated negatively with perceived physical and mental health, highlighting how chronic sleep disruption can erode both individual well-being and aspects of relational functioning (Hasler & Troxel, 2010; Troxel et al., 2009). In the control group, these patterns were less complex: relationship quality correlated mainly with satisfaction and cohesion, and sleep quality had weaker associations, reinforcing the specific burden of OSA on partners.

Across both groups, affective expression, satisfaction, and cohesion were positively associated with perceived health, underscoring the potential buffering role of emotional intimacy in mitigating health-related stressors (Revenson & DeLongis, 2010; Røsand et al., 2012).

In conclusion, partners of OSA patients exhibit lower sleep quality, poorer health perception, and reduced couple functioning compared to controls. While these findings should be interpreted cautiously due to the cross-sectional design, the absence of poor sleepers in the control group, and exploratory relational classifications, they emphasize the systemic nature of OSA, affecting both patients and partners. Emotionally responsive and cohesive relationships may serve as important resources, potentially buffering stress and supporting resilience. Future longitudinal research, more heterogeneous controls, and clearer operational definitions of relational categories are needed to disentangle condition-specific effects from sampling influences. Overall, these results reinforce the importance of conceptualizing OSA as a relational phenomenon, warranting assessment and intervention at the level of the couple, in addition to the patient.

### Limitations

Several limitations should be acknowledged when interpreting the present findings. First, the cross-sectional design precludes any causal inference regarding the directionality of the associations observed among sleep quality, relationship functioning, and perceived health. Although significant correlations emerged, it remains unclear whether poorer sleep leads to relational strain and reduced well-being, whether relational difficulties exacerbate sleep disturbances, or whether these processes are reciprocally reinforced over time.

Second, the sample size was relatively modest and predominantly female, which may limit the generalizability of the findings. In addition, the control group unexpectedly included only participants classified as “good sleepers,” despite sleep quality not being an inclusion criterion. Community-based samples generally include a non-negligible proportion of individuals with poor sleep quality thus, the present control group may not fully represent the variability of sleep quality observed in the general population. The observed differences in sleep quality should not be interpreted exclusively as reflecting the impact of OSA or CPAP treatment on partners, but also as potentially influenced by the unusually favorable sleep profile of the control group. Future studies should recruit more heterogeneous and representative comparison groups, including controls with different types of sleep quality, to better disentangle condition-related effects from sampling characteristics.

Moreover, the absence of correction for multiple statistical tests represents an additional limitation. This analytic choice increases the risk of type I error and consequently weakens the generalizability of the significant findings. Accordingly, the results should be interpreted with caution and considered as important preliminary evidence that may guide future studies using larger samples, confirmatory designs, and appropriate correction procedures for multiple comparisons.

Third, sleep quality was assessed exclusively through self-report measures (PSQI), without objective sleep recordings. Although the PSQI is widely validated and has demonstrated good internal consistency in the present study, subjective sleep perception may not fully capture the complexity of sleep disturbances in partners of patients with OSA. Similarly, relationship functioning was assessed using self-report questionnaires, which may be influenced by response bias or social desirability.

Furthermore, the categorization of couple functioning (e.g., “functional distress”) was exploratory and not based on clinically validated cut-offs specifically established for relational typologies. While this approach allowed for a descriptive differentiation of relational profiles, these classifications should be interpreted with caution and considered preliminary.

Despite these limitations, the study offers several important contributions. It addresses an underexplored population of partners of CPAP-treated OSA patients within a dyadic framework, integrating sleep quality, relationship functioning, and health-related QoL in a single model. The use of validated instruments (PSQI, DAS, SF-36) and the comparison with a demographically similar control group strengthen the methodological rigor. Most importantly, the findings contribute to a growing body of literature conceptualizing OSA as a “shared” condition embedded within a relational system. By highlighting the interconnected nature of sleep, health perception, and couple functioning, this study provides clinically relevant insights that support the development of couple-centered assessment and intervention strategies in sleep medicine.

### Conclusions and Future Directions

In conclusion, our comparative analysis highlights a general decline across multiple domains, including sleep quality, health perception, and relationship functioning among partners of patients with OSAS relative to the control group. Although these findings must be interpreted cautiously in light of methodological limitations, the exploratory nature of relational classifications, and the cross-sectional design, they contribute to a growing body of evidence suggesting that obstructive sleep apnea should not be conceptualized solely as an individual clinical condition. Rather, OSA appears to extend its impact to the relational sphere, shaping partners’ well-being and the overall quality of couple functioning.

The observed pattern underscores the interconnected nature of sleep, relational quality, and perceived health. While the present design does not allow causal conclusions, the findings suggest that relational functioning and individual psychological well-being are closely intertwined. In this context, emotionally responsive and cohesive relationships may represent an important relational resource, potentially buffering stress and fostering resilience within the dyad.

From a clinical perspective, these results support the importance of adopting a dyadic approach in the management of OSAS. Including partners in assessment procedures, psychoeducation, and CPAP adherence strategies may enhance not only patient outcomes but also relational stability and mutual well-being.

Future research should employ longitudinal designs to clarify the directionality of associations between sleep disturbances and couple functioning, recruit more heterogeneous and representative control samples, and further refine the operationalization of relational profiles. The integration of objective sleep measures and dyadic-level analyses would also strengthen the understanding of how chronic sleep disruption affects couple dynamics over time. Advancing this line of inquiry may ultimately inform more comprehensive, couple-centered models of care in sleep medicine.

## Data Availability

The data that support the findings of this study are available from the corresponding author upon reasonable request. Materials: The materials used in this study are described in the Method section.

## Preregistration

No preregistration exists for this study.

## Reporting Guidelines

APA Guidelines for Psychological Assessment and Evaluation were followed [available link: APA Guidelines for Psychological Assessment and Evaluation]
